# Vascularity of Tooth-Bone

**Published:** 1842-09

**Authors:** 


*Vascularity of Tooth-bone.*—We were shown a short time since, by Dr. Maynard, of Washington, a molar tooth, the bone of which, externally and internally, extending more than one-third the way through the parieties of its roots, was beautifully suffused with red. This circumstance conclusively establishes the doctrine that the investing as well as the lining membrane of a tooth, contributes to the vitality of tooth-bone. We hope to be able, in some future number, to present our readers with a drawing of it.

